# PW03-027 - CASP1 variants and live cell imaging

**DOI:** 10.1186/1546-0096-11-S1-A253

**Published:** 2013-11-08

**Authors:** S Rabe, MC Heymann, S Russ, S Winkler, J Roesler, A Rösen-Wolff, SR Hofmann

**Affiliations:** 1 Department of Pediatrics, Technical University Dresden, Medical Center Carl Gustav Carus, Dresden, Germany

## Introduction

Patients with unexplained recurrent febrile episodes and *CASP1* variants suffer from systemic sterile inflammation despite reduced secretion of IL-1ß. As previously demonstrated by our group *CASP1* variants lead to reduced enzymatic activity of procaspase-1 by destabilizing the tertiary structure of the caspase-1 tetramer. A possible explanation for an alternative pro-inflammatory pathway has been provided by Lamkanfi and colleagues indicating an association between enzymatically inactive procaspase-1 and receptor interacting protein kinase 2 (RIP2) leading to NF-kB activation.

## Objectives

The objective of this project is the identification of possible subcellular mechanisms how *CASP1* variants interfere with the IL-1β production or release and lead to the activation of alternative pro-inflammatory pathways.

## Methods

Using confocal microscopy, in vivo live cell imaging and an in situ proximity ligation assay we analyzed the subcellular distribution of procaspase-1 wildtype and mutants as well as the interaction with RIP2 in naïve or virally transduced THP-1 cells.

## Results

THP-1 cells were virally transduced with GFP- or mCherry fusion proteins of procaspase-1 wildtype and variant *CASP1-L*265S. Procaspase-1 activation, initiated by the assembly of multiprotein complexes (inflammasomes), was induced by stimulation with LPS and Nigericin. First results suggest disturbed microvesicle shedding from *CASP1-L*265S expressing cells after administration of Nigericin. In addition to live cell imaging, the interaction of procaspase-1 and RIP2 has been studied in vitro in naïve THP-1 cells using antibody labeling and proximity ligation assay showing a time dependency after LPS stimulation.

## Conclusion

This result suggests a possible influence of procaspase-1 variants on plasma membrane properties, pyroptosis and the release of microvesicles.

## Disclosure of interest

None declared

